# Supplementary material for: Disulfide stabilization reveals conserved dynamic features between SARS-CoV-1 and SARS-CoV-2 spikes
Source: Life Sci Alliance. 2023 Jul 4;6(9):e202201796. doi: 10.26508/lsa.202201796 (PMC10320017; doi:10.26508/lsa.202201796)
Supplement: Supplementary file 1 [file LSA-2022-01796_TableS1.doc]

**Table S1. Cryo-EM data collection, refinement and validation statistics**

|  | S/x1 locked-1  (EMDB-34417)  (PDB: 8H0X) | S/x1 locked-112  (EMDB-34418)  (PDB: 8H0Y) | S/x1 locked-122  (EMDB-34419)  (PDD: 8H0Z) | | S/x1 locked-2  (EMDB-34420)  (PDB: 8H10) | | S/x1 closed  (EMDB-34421)  (PDB: 8H11) |
| --- | --- | --- | --- | --- | --- | --- | --- |
| **Data collection and processing** |  | | | | | | |
| Magnification | 81000  300  50  -0.8~-2.0  1.095 | | | | | | |
| Voltage (kV) |
| Electron exposure (e^–^/Å^2^) |
| Defocus range (μm) |
| Pixel size (Å) |
| Symmetry imposed | C3 | C1 | | C1 | | C3 | C1 |
| Initial particle images (no.) | 4604239 | 4604239 | | 4604239 | | 4604239 | 4604239 |
| Final particle images (no.) | 58285 | 91477 | | 70911 | | 22532 | 504118 |
| Map resolution (Å)  FSC threshold | 2.57  0.143 | 2.85  0.143 | | 2.99  0.143 | | 2.99  0.143 | 2.72  0.143 |
| Map resolution range (Å) | 2.46-5.18 | 2.74-4.74 | | 2.87-4.65 | | 2.85-4.74 | 2.55-4.92 |
| **Refinement** |  | | | | | | |
| Initial model used (PDB code) | 7XTZ | 7XTZ/7XU2 | | 7XTZ/7XU2 | | 7XU2 | 5X58 |
| Model resolution (Å)  FSC threshold | 2.7  0.5 | 2.9  0.5 | | 3.0  0.5 | | 3.1  0.5 | 2.8  0.5 |
| Map sharpening *B* factor (Å^2^) | -26 | -30 | | -29 | | -34 | -48 |
| Model composition  Non-hydrogen atoms  Protein residues  Ligands | 24804  3078  777 | 24940  3086  833 | | 24797  3078  776 | | 24882  3078  861 | 23477  2932  560 |
| *B* factors (Å^2^)  Protein  Ligand | 46.20  61.70 | 64.68  80.90 | | 65.79  81.68 | | 54.21  78.21 | 38.19  74.64 |
| R.m.s. deviations  Bond lengths (Å)  Bond angles (°) | 0.004  0.988 | 0.003  0.73 | | 0.006  0.725 | | 0.007  1.054 | 0.004  0.665 |
| **Validation** |  | | | | | | |
| MolProbity score  Clashscore  Poor rotamers (%) | 1.19  3.31  0.26 | 1.10  2.14  0.3 | | 1.20  4.17  0.3 | | 1.20  2.98  0.34 | 1.04  2.50  0 |
| Ramachandran plot  Favored (%)  Allowed (%)  Disallowed (%) | 97.69  2.31  0 | 97.46  2.54  0 | | 97.98  2.02  0 | | 97.43  2.57  0 | 97.97  2.03  0 |

|  | S/x2 closed  (EMDB-34422)  (PDB: 8H12) | S/x2 locked-2  (EMDB-34423)  (PDB: 8H13) | S/x3 locked-1  (EMDB-34424)  (PDB: 8H14) |
| --- | --- | --- | --- |
| **Data collection and processing** |  | | |
| Magnification | 45000x  200  62  -0.8~-2.4  0.88 | | |
| Voltage (kV) |
| Electron exposure (e^–^/Å^2^) |
| Defocus range (μm) |
| Pixel size (Å) |
| Symmetry imposed | C3 | C3 | C3 |
| Initial particle images (no.) | 967643 | 967643 | 82693 |
| Final particle images (no.) | 82882 | 192457 | 22958 |
| Map resolution (Å)  FSC threshold | 3.96  0.143 | 3.37  0.143 | 3.28  0.143 |
| Map resolution range (Å) | 3.79~9.19 | 3.26~6.92 | 3.11~10.00 |
| **Refinement** |  | | |
| Initial model used (PDB code) | 5x58 | 7XU2 | 7XTZ |
| Model resolution (Å)  FSC threshold | 4.04  0.5 | 3.45  0.5 | 3.38  0.5 |
| Map sharpening *B* factor (Å^2^) | -137 | -105 | -39.5 |
| Model composition  Non-hydrogen atoms  Protein residues  Ligands | 22233  2793  378 | 24330  3036  630 | 24762  3075  777 |
| *B* factors (Å^2^)  Protein  Ligand | 43.73  86.83 | 27.86  55.30 | 25.88  46.88 |
| R.m.s. deviations  Bond lengths (Å)  Bond angles (°) | 0.004  0.758 | 0.004  1.096 | 0.003  0.597 |
| **Validation** |  | | |
| MolProbity score  Clashscore  Poor rotamers (%) | 1.58  3.92  0.08 | 1.21  2.27  1.03 | 1.05  2.03  0 |
| Ramachandran plot  Favored (%)  Allowed (%)  Disallowed (%) | 93.91  6.09  0 | 96.79  3.21  0 | 97.63  2.37  0 |

|  | S/native low-pH closed  (EMDB-34425)  (PDB: 8H15) | S/native low-pH open  (EMDB-34426)  (PDB: 8H16) |
| --- | --- | --- |
| **Data collection and processing** |  | |
| Magnification | 45000x  200  60  -0.8~-2.5  0.88 | |
| Voltage (kV) |
| Electron exposure (e^–^/Å^2^) |
| Defocus range (μm) |
| Pixel size (Å) |
| Symmetry imposed | C3 | C1 |
| Initial particle images (no.) | 1375445 | 1375445 |
| Final particle images (no.) | 201293 | 308387 |
| Map resolution (Å)  FSC threshold | 3.07  0.143 | 3.28  0.143 |
| Map resolution range (Å) | 2.95~9.13 | 3.07~9.02 |
| **Refinement** |  | |
| Initial model used (PDB code) | 5X58 | 5X5B |
| Model resolution (Å)  FSC threshold | 3.07  0.5 | 3.28  0.5 |
| Map sharpening *B* factor (Å^2^) | -68.5 | -63.5 |
| Model composition  Non-hydrogen atoms  Protein residues  Ligands | 22571  2890  392 | 20534  2630  0 |
| *B* factors (Å^2^)  Protein  Ligand | 22.64  49.45 | 27.80  0 |
| R.m.s. deviations  Bond lengths (Å)  Bond angles (°) | 0.003  0.72 | 0.004  0.82 |
| **Validation** |  | |
| MolProbity score  Clashscore  Poor rotamers (%) | 1.22  3.11  0.12 | 1.45  4.44  0.44 |
| Ramachandran plot  Favored (%)  Allowed (%)  Disallowed (%) | 97.41  2.59  0 | 96.47  3.53  0 |
